# Estimating The Annual Abortion Rate in Kerman, Iran: Comparison of Direct, Network Scale-Up, and Single Sample Count Methods

**DOI:** 10.22074/ijfs.2019.5721

**Published:** 2019-07-14

**Authors:** Maryam Zamanian, Farzaneh Zolala, Ali Akbar Haghdoost, Mohammad Reza Baneshi

**Affiliations:** 1 Department of Epidemiology, School of Health, Arak University of Medical Sciences, Arak, Iran; 2 Modeling in Health Research Center, Institute for Futures Studies in Health, Kerman University of Medical Sciences, Kerman, Iran; 3 Social Determinants in Health Research Center, Institute for Futures Studies in Health, Kerman University of Medical Sciences, Kerman, Iran; 4 HIV/STI Surveillance Research Center, and WHO Collaborating Center for HIV Surveillance, Institute for Futures Studies in Health, Kerman University of Medical Sciences, Kerman, Iran

**Keywords:** Abortion, Indirect, Network Scale-Up, Single Sample Count, Kerman

## Abstract

**Background:**

Abortion is a sensitive issue surrounded by social, cultural and religious stigmata. Therefore, estimation of its prevalence involves methodological challenges. The aim of this manuscript is to estimate the abortion prevalence, stratified by type, using a direct and two indirect methods.

**Materials and Methods:**

This cross-sectional study was done in 2016, we recruited 1020 women aging 18-49 years. Three methods were applied to estimate the abortion prevalence: direct question, network scale-up (NSU), and single sample count (SSC). In the direct method, to guarantee anonymity, data were collected by means of a self-administered questionnaire. In other methods, data were collected through gender-matched street-based inter- views.

**Results:**

The annual rate of abortion estimated by direct and NSU methods were respectively 29 (10 intentional, 4 therapeutic and 15 spontaneous) and 23 (9 intentional, 3 therapeutic, and 11 spontaneous) per 1000 women aging 18-49 years. The annual rate of intentional abortion estimated based on SSC method was higher (15 per 1000 women) than other methods.

**Conclusion:**

The present estimates are higher than previously reported ones. The results of three methods more or less supported each other confirming the internal validity of our estimates.

## Introduction

 Abortion is an important contributing factor to women’s health and it could even result in mother’s death. Although in many societies, abortion has been associated with legal restrictions as well as social, cultural and religious stigmata (1-3), placing legal limitations has not reduced its prevalence. In contrast, legal restrictions have increased the number of women who seek for clandestine and unsafe abortion in illegal and underground abortion centers to terminate their unintended pregnancies (4). Almost all (i.e. 98%) unsafe abortions, which is regarded as the third cause of maternal death (5), occur in developing countries; nevertheless, in contrast to other causes of women’s death, all complications and deaths caused by unsafe abortions, are preventable (6, 7).

International Journal of Fertility and Sterility Vol 13, No 3, October-December 2019, Pages: 209-214 Legal restrictions and stigmata around abortion make it invisible, as those who had abortion are not willing to disclose it. This in turn might lead to inaccurate data on the annual number of abortions (8, 9). This issue goes beyond the culture and law and even in communities with legal abortion policy, underreporting accounts for 70% of cases (10). A similar issue is likely to happen for spontaneous abortions in developing countries due to failure of registration systems (11). Therefore, the available data might only present the tip of the iceberg (12). It should be also noted that policy makers need reliable data for appropriate decision making.

Due to the social stigma and legislations, direct estimation of abortion rate with face-to-face interviews might result in under-estimation of the true prevalence. In this context, indirect methods were proposed for estimation of the size of hidden and stigmatized subpopulations (13). Among indirect methods, network scale-up (NSU) is an appealing method that does not need direct contact with the target population (14-16). In NSU, a random sample of general population is recruited and interviewed to count the number of individuals with a given characteristics (for example those who had abortion) among their social network. The NSU method is based on the principle that the proportion of people that participants know from the target population, directly corresponds to the actual size of the community (17-19). This method preserves anonymity as respondents reply on behalf of their network rather than themselves. It was shown that NSU is a valid tool for estimation of the size of hidden groups (20).

Single sample count (SSC) technique is another efficient indirect method. In this method, a list of statements including several insensitive statements with certain distribution plus a sensitive one is given to the participants (21). Here, the participant is asked to determine how many of statements are true about herself /himself. In Iran, a country with Islamic State, which is located in the Middle East, intentional abortion is strictly prohibited. Hence, underreporting and misclassification of abortions is high in usual health reports. From 2012 onwards, the overall policy of the country has changed to increase fertility rate, which itself could impose further limitations on performing abortion.

Results of the studies which were designed to estimate intentional abortion rate in Iran are inconsistent, ranging from 1 to 20%. In a meta-analysis study, the annual prevalence was estimated to be 8.9 per 1000 women of fertile age (22). We conducted the present study in Kerman city located in south east of Iran, and aimed to estimate the abortion rate, and compare performance of direct and indirect methods. 

## Materials and Methods

### Sampling process

This cross-sectional study was conducted in Kerman, south east of Iran. In this study, 1020 women aging 18- 49 years were selected through street-based multistage sampling proportional to the age distribution of women in the 2011 census. At the first step, the city was classified into three categories based on socioeconomic status (SES): high, medium and low. To do so, we asked for the governor’s office experts' opinion. Secondly, 5 regions were selected from each SES category. Finally, from each of 15 regions, 68 women were recruited through the convenience sampling method in streets. We adopted street-based sampling, as our previous experiences showed that in case of sensitive issues, other sampling schemes such as household or telephone-based methods do not work.

The eligible participants were women aging 18-49 years, who had been living in Kerman for the past five years and verbally consented to participate in the study. Data was collected through a structured, face-to-face interview both in the morning and evening times, performed by trained female interviewers. The proposal of this study was approved by the Ethical Committee of Kerman University of Medical Sciences (ir.kmu. rec.1394.223).

### Network scale-up estimation

In the first section of the questionnaire, general explanations about the study and the aims were provided for participants. In the next section, NSU questions were asked “how many women do you know in the city of Kerman, who experienced an abortion within the last year?” In order to minimize the recall bias, this question was stratified. We asked participants to tell the number of such individuals they know among their relatives, husband relatives (in case she was married), and acquaintances (involving neighbor, friend, colleague, etc.). These questions were followed by questions on type of abortion (intentional, therapeutic, or spontaneous), and age of mother. The standard definition of ‘know’ was as follows: “you know them by name and face, and have had at least one contact through phone, mail, or meeting in person within the past two years, and are able to contact them at any time through one of the above-mentioned methods” (23). To help participants to distinguish different types of abortion, a short and simple description of the three types of abortions was provided in the questionnaire as follows: spontaneous abortion is an abortion which occurs without intervention. Therapeutic abortion is permitted due to fetal abnormalities or to protect the mother’s life. Intentional abortion is elective termination of pregnancy without medical justification.

The first requirement of using NSU is knowing the network size (C) of the participants. In this study we needed the number of women at reproductive age known by residents of Kerman. This had been assessed previously (24) and it had been shown that, on average, women aging 18- 49 years in Kerman know 111 women aging 18-49 years. 

The following formulas were applied to estimate the crude size of abortion and its standard error (SE): 

Formula (1) $$e_{j}=\frac{\Sigma m_{\mathrm{ij}}}{\Sigma c_{i}}t$$

Formula (2) $$\mathrm{se}=\sqrt{\frac{e_{j}}{\Sigma c_{i}}t}$$

Where i and j stand for respondent and hidden group (i.e. abortion), respectively, m is the number of abortions known by each respondent, c is the average network, and t is the total population of 18-49 year old females living in Kerman city, which was about 155,644 according to the latest Iranian census. 

It is possible that those who had abortion, do not reveal it to their network members. This is known as visibility bias. We already designed a study to measure visibility of different types of abortion. It was determined 11, 70, and 60%, for intentional, therapeutic, and spontaneous abortion, respectively (25). Crude estimations were divided by these visibility rates to provide the adjusted size estimates. 

### Single sample count estimation

In the SSC section, a five-statement list, including four insensitive questions plus a sensitive question on intentional abortion, was given to each participant. The prevalence of each of insensitive questions in the society was 50%. Then, the participant was required to determine how many statements were true for her case. We emphasized that it is not necessary to explain which ones, but simply declare the number of statements that apply to her. In this study, we only estimated the prevalence of intentional abortion. The five statements were as follows: i. My national ID card number is even, ii. My date of birth is in the first 15 days of the month, iii. The year of my birth is even, iv. I was born in the first six month of the year, and v. I had intentional abortion within the past year. 

The probability of a ‘yes’ response to each of non- sensitive items was 50%. We assumed that each of them follows a binomial distribution with 50% probability of success. Therefore, the expected mean of replies to four insensitive questions was two. Therefore, any deviation from two can be attributed to the sensitive statements. The formulas used for calculating prevalence rate and its confidence interval are given below. Here, λ and n show the number of ‘yes’ replies and sample size, respectively. 

Formula (3) d=(λn)-2

Formula (4) $$\lambda \pm Z{\left(0.95\right)}^{*}\sqrt{\left(n^{*}(1+d^{*}(1-d))\right)}$$

### Direct estimation

Finally, at the last section, in the direct method, the participants were provided with a questionnaire about their own experience of abortion within the preceding year. This was conducted regardless of participants’ marital status. Moreover, the questions were self-administrated and the completed questionnaires were collected through a ballot box to be more comfortable for the participants and increase the accuracy of responses. Data were analyzed using stata version 11 and Excel software. 

## Results

Among 1451 female who were residents of Kerman and aged 18-49 years and were invited to join the study, 1020 consented to participate, giving a response rate of 70.3%. The youngest and the oldest participants were 18, and 49 years old, respectively. The mean (SD) age of the participants was 30.84 year (8.57). About two-third of the participants were married, and nearly half of them had university educations (i.e. more than 12 years of education). Moreover, about 30% of them were employed. We asked married women to provide demographic characteristics of their husbands. Nearly one third of husbands had university educations, and more than half of them were self-employed (Table 1).

In total, 41.8% of participants did not know any woman who had an abortion in the past year. The mean (± SD) number of abortions known by respondents was 1.07 (± 1.55). Poisson regression model revealed that married and widowed subjects were respectively 39 and 12% more likely to reveal abortion than single participants. Those in age groups of 25-34 and 18-24 years, in comparison with those aged 35-49 years, were respectively 72 and 57% more likely to report abortion cases in their network. Employees and self-employed women were respectively 43 and 28% more likely to report abortion. 

**Table 1:** Demographic characteristics of participants


Variable	Category	n (%)

Age (Y)	18-24	301 (29.5)
	25-34	391 (38.3)
	35-49	328 (32.2)
Marital status	Single	354 (34.7)
	Married	643 (63.0)
	Divorced/widowed	23 (2.3)
Job	Housewife	465 (45.6)
	Employee	159 (15.6)
	Student	195 (19.1)
	Self-employed	158 (15.5)
	Retired	7 (0.7)
	Unemployed	36 (3.5)
Education	≤9 years	136 (13.4)
	12 years	392 (38.4)
	12-16 years	403 (39.5)
	≥18	89 (8.7)
Husband’s job	Employee	196 (30.5)
	Worker	54 (8.4)
	Self-employed	357 (55.5)
	Retired	26 (4)
	Un-employed	10 (1.6)
Husband’s education	≤9 years	158 (24.6)
	12 years	271 (42.1)
	12-16 years	165 (25.7)
	≥18	49 (7.6)


As summarized in Table 2, NSU estimates for intentional, therapeutic, and spontaneous abortions were 9, 3, and 11 per 1000 women of reproductive ages. In SSC method, the average positive answers for five-item list were 2.015. This suggested an annual prevalence of intentional abortion at 15 per 1000 women of reproductive ages. The estimates of direct method for three types of abortion namely intentional, therapeutic, and spontaneous were 10, 4, and 15 abortions per 1000 women of reproductive ages, respectively.

**Table 2:** The annual abortion rate determined by the three methods


Type of abortion	Direct% (CI 95%)	NSU% (CI 95%)	SSC% (CI 95%)

Intentional	0.98 (0.38-1.58)	0.9 (0.73-1.1)	1. 5 (0-7.6)
Therapeutic	0.39 (0.006-0.77)	0.29 (0.25-0.33)	
Spontaneous	1.47 (0.73-2.21)	1.12 (1.04-1.2)	


NSU; Network Scale UP, SSC; Single Sample Count, and CI; Confidence Interval.

## Discussion

In this study, using the NSU and direct method, the annual rate of abortion per 1000 women aging 18-49 years was calculated to be about 23 (9 intentional, 3 therapeutic, and 11 spontaneous abortion) and 29 (10 intentional, 4 therapeutic, and 15 spontaneous abortion), respectively. Also, using SSC method, intentional abortion was estimated to be 15 per 1000 women aging 18-49 years.

The results of direct and NSU methods were fairly close with overlap in their confidence intervals. The estimates of the direct method were slightly higher than those of the NSU method. This might be due to this issue that since for direct estimation, a self-administered questionnaire at the end of interview was submitted to the respondents and the forms were returned through a ballot box, the anonymity the response was maximized. This indicates that use of direct methods with consideration of methodological issues can provide useful statistics. It also implies the usefulness of NSU. In comparison with direct and item counts methods, the confidence intervals of NSU method were narrower. Within the NSU method, each person responds about the intended behavior of the whole network members rather than one individual. Therefore, the sample size required for NSU studies is much smaller than that of direct methods. 

We applied SSC method only for intentional abortion. The SSC estimate was higher than those of the other methods and its confidence interval was wider, which might be due to the nature of this method (26).

Our estimate for intentional abortion (9-15 cases per 1000 women aging 18-49 years) was slightly higher than that reported by two national studies in Iran (12, 27). We should mention that comparison of our results with previous studies is not simple due to methodological differences. For example, face-to-face interview was the dominant data collection method in previous studies. Demographic and Health Survey data of 2000, estimated the annual rate of intentional abortion to be 7.5 per 1000 married women aging 15-49 years (2). Also, meta-analysis of manuscripts published by 2012, estimated an annual rate of 8.9 per 1000 married woman aging 15-44 years (22). The difference is even larger, as the denominator in our calculations included all women aging 18 to 50 years, while previous studies provided estimates just for married women of reproductive ages. 

The denominator in our study included all women of reproductive ages, in order to make our results comparable with those of WHO and studies conducted in other countries (28). We should mention that the proportion of single cases in our sample was the same as that of the population.

Zare and Dastouri (29) recruited 550 married women aging 15 to 49 years, who referred to two governmental clinics in Shiraz, south of Iran. They reported a life time intentional abortion rate of 29 cases per 1000 married women aging 15-49 years. This value is higher than our estimate, as they provided life time not annual prevalence, and they considered a small population.

Nojomi et al. (30) carried out a study on 2470 ever- married women using the direct method and face-to-face interview. They estimated an annual intentional and spontaneous abortion rate of 27 per 1000 women aging 15-55 years in Tehran. 

The worldwide estimate of intentional abortions per 1000 women aging 15-44 years is 35 (27 and 37 in developed and developing countries, respectively). This corresponded to 25% of pregnancies. Globally, married and single women account for 73 and 27% of abortions, respectively. The range of intentional abortion in Asia was reported to be 35-37, as well (28). These data suggested a lower prevalence of abortion in Iran than world statistics. Less pre-marital sexual relationships could be one of the reasons. Even pregnancy during “Aghd” period (when marriage contract is approved by the authorities but the couple does not yet share an accommodation) is against social norms. In addition to that, in some traditional Iranian families, being virgin on the wedding night, which is certified by a gynecologist, is an important custom. Even after marriage, the rate of intentional abortion is lower than that of other countries mainly due to religious believes, legal restrictions, punishments, and lack of access to standard health centers to provide services. 

Based on the world statistics, more than half of the unplanned pregnancies (about 57%) ends in intentional abortion (4). We believe that, due to issues noted above, the corresponding figure in Iran is much lower. Results of a recent meta-analysis in 2012 suggested that the prevalence of unplanned pregnancy in Iran is about 28% (22). Based on another study unwanted birth accounts for 20% of all born children (31). This indicates that in Iran, most of unplanned pregnancies result in unwanted birth.

Although abortion is considered against social norms and standard services are not available for that in Iran, we believe that its rate is still considerable. This is alarming and policy makers should be informed to explore for possibility of new legislations. Women need more assistance and guide from health care providers to make better decisions in their reproductive life. Moreover, providing enough resources for reproductive health services for them is vital (32).

One of the limitations of our study was that, due to ethical issues, we could not recruit those aged under 18. Moreover, street-based interviews does not guarantee access to a random sample. However, it was a trade-off between representativeness of the sample and accuracy of replies. On the other hand, our study had several strengths. It was the first study that compared performance of direct and indirect methods in estimation of abortion rate. We showed that even direct methods are applicable, if methodological issues are concerned and anonymity is preserved. We provided an updated figure for the abortion situation in Iran.

## Conclusion

Estimates derived in our study are alarming and flashes the need for new legislations. The results of three methods are close confirming the internal validity of methods and methodologies. While direct method with methodological considerations might still provide an acceptable estimate, NSU method has practical appeal as it requires a much smaller sample size in sensitive issues with relatively low prevalence. In addition, it is possible to estimate size of several hidden groups in one study. Furthermore, these indirect methods might be useful and are suggested in estimating other sensitive issues through increasing the response and honesty rate. In addition, such methods enjoy from some advantages, like cost-effectiveness, quickness, and simplicity in performance and analysis, which make them an appropriate tool in low and middle income countries, where an accurate registration system is lacking. 
